# Barriers to Regular Eye Examination in Individuals with Diabetes at a Tertiary Diabetes Centre in Jordan: A Cross-Sectional Study

**DOI:** 10.3390/ijerph23020147

**Published:** 2026-01-24

**Authors:** Yazan J. Albakri, Fatema A. Aldabbagh, Hashem M. Sabbagh, Mohammad K. Khashman, Oraib Farahid, Rasha M. Ali, Almutez M. Gharaibeh

**Affiliations:** 1 Jordan University Hospital, Amman 11942, Jordan; 2 National Center for Diabetes, Endocrinology and Genetics, Amman 11942, Jordan; 3 Faculty of Medicine, The University of Jordan, Amman 11942, Jordan

**Keywords:** diabetic retinopathy, diabetes mellitus, health services accessibility, health knowledge, attitudes, practice, healthcare disparities, ophthalmic examination, screening barriers, patient adherence

## Abstract

**Highlights:**

**Public health relevance—How does this work relate to a public health issue?**

- Diabetes is a worldwide disease that affects many people and could cause many complications such as diabetic retinopathy.

**Public health significance—Why is this work of significance to public health?**

- This study highlights the importance of regular eye examination in the early recognition of diabetic retinopathy.
- The study aims to identify the barriers that affect adherence to regular diabetic retinopathy screening among people with diabetes. Although these barriers have been studied individually in other countries, this research combines them together.

**Public health implications—What are the key implications or messages for practitioners, policy makers, and/or researchers in public health?**

- The most prevalent barriers to diabetic retinopathy screening in Jordan are modifiable and rooted in low awareness and accessibility.
- Directing more focus towards modifiable barriers could help facilitate and ensure the early detection of diabetic retinopathy.

**Abstract:**

Background: Diabetic retinopathy is a leading cause of vision impairment and a significant complication of diabetes mellitus, especially in low- and middle-income countries. This study aimed to identify the barriers affecting diabetic retinopathy screening among people with diabetes mellitus. Methods: This cross-sectional study was conducted between April and October 2024 at the National Center for Diabetes, Endocrinology and Genetics. Data collection was performed using a structured, validated electronic questionnaire adapted from previous studies. Sample size calculation was carried out before data collection. Data were collected using a structured electronic questionnaire. A total of 998 responses were included in the study. The collected data incorporated sociodemographic characteristics, diabetes history, screening practices, and reported barriers. Descriptive and categorical data analyses were performed. Results: Of 998 participants, 82% were over 50 years old, 79% had type 2 diabetes mellitus, and 30% had never had an eye examination. Diabetic retinopathy was diagnosed in 12%. The main barriers to regular attendance among those previously screened (699) were as follows: lack of awareness of its importance (11%), believing that being asymptomatic made screening unnecessary (19%), and transportation difficulties (14%). Among those never screened (299), 56% lacked awareness, 62% believed being asymptomatic negates the need for screening, and 13% faced transportation difficulties. Age > 50 years, higher educational level, availability of health insurance, longer duration of diagnosis of diabetes mellitus, and HbA_1c_ > 7% were significantly associated with prior screening (*p* < 0.05). Conclusions: Public health strategies should enhance the education provided to people and physician–person communication and remove logistical obstacles to improve screening compliance.

## 1. Introduction

Diabetes mellitus is a global public health problem increasing in prevalence and impact, especially in middle- and low-income countries [1]. The number of individuals with diabetes is estimated to grow annually by 2.2%, which is nearly double the annual growth rate of the global population [2]. The Middle East and North Africa (MENA) region has the highest regional incidence at 84.7 million (17.6%), and this is expected to rise significantly to 163 million by 2050 [3]. In Jordan, the overall age-standardised rates of individuals diagnosed with diabetes rose from 13% in 1994 to 23.7% in 2017 [4].

Diabetic retinopathy, a disease that affects the microvasculature of the retina, is a major complication of diabetes [5]. There are two types of diabetic retinopathy: non-proliferative diabetic retinopathy (NPDR) and proliferative diabetic retinopathy (PDR). Another complication of diabetes is diabetic macular oedema, which is defined by the Early Treatment Diabetic Retinopathy Study (ETDRS) as a retinal thickening or the presence of hard exudates within 1 disc diameter of the macula [6]. PDR and diabetic macular oedema are the main complications that result in vision-threatening diabetic retinopathy (VTDR). Similarly, diabetic retinopathy and VTDR are among the leading causes of blindness worldwide, with recent studies predicting that the number of adults with diabetic retinopathy and VTDR will rise from 103.12 million and 28.54 million in 2020 to 160.50 million and 44.82 million by 2045, respectively [7]. A combination of screening, good blood glucose management, early detection, and treatment of any retinal changes reduces the risk of VTDR [8,9].

These conditions, occurring individually or in combination, are asymptomatic in the early stages. Therefore, they are best recognised early on through retinal examination [10]. A unified screening programme would not only detect changes early, but could reduce the percentage of diabetic retinopathy positive results [11]. The recommendations for the initial examination are as follows: 5 years after diagnosis for type 1 diabetes and at the time of diagnosis for type 2 diabetes [12]. For follow-up, if there is no diabetic retinopathy, every 1 to 2 years; if mild NPDR, every 6 to 12 months; if moderate NPDR, every 3 to 6 months; if severe NPDR, once every less than 3 months; if PDR, at least once every month [8].

Several studies have examined the barriers to diabetic retinopathy screening among people with diabetes. Some of these barriers are as follows: sociodemographic disparities, such as race and gender, difficulties obtaining time off work/study to attend appointments, anxiety about screening results, younger age groups, lower levels of income and education, difficulty accessing screening services and scheduling appointments, lack of awareness of the reasons for screening, time required for registration, and a lack of readily available information in their local area/practice [13,14,15,16].

The study aims to identify the barriers that affect adherence to regular diabetic retinopathy screening among people with diabetes. Although these barriers have been studied individually in other countries, this research integrates multiple previously reported barriers within a single framework in the Jordanian population, addressing an important gap in the national literature. Based on the findings, policy-relevant strategies include enhancing patient education at the time of diabetes diagnosis, improving physician–patient communication about the importance of eye health, and mitigating practical barriers such as transportation challenges and limited appointment accessibility, which together may strengthen adherence to the recommended screening schedules.

## 2. Materials and Methods

This cross-sectional study, conducted at the National Center for Diabetes, Endocrinology and Genetics (NCDEG), examined the barriers to ophthalmic screening among the Jordanian population. Institutional Review Board (IRB) approval was obtained in April 2024. The IRB approval number was 1/2024.

The planned sample size was based on estimating the prevalence of never having had an ophthalmic examination among people with diabetes. Using the single-proportion formula (*n* = Z^2^·p·(1 − p)/d^2^) with Z = 1.96 for 95% confidence, an anticipated prevalence *p* = 0.30 (based on pilot data and prior regional studies) [17], and a precision (d) of 0.03 (3 percentage points), the required sample size is approximately 897. To allow for incomplete responses and subgroup analyses, the sample size was inflated by 10%, giving a target of around 987 participants. A total of 1016 participants with diabetes from NCDEG’s outpatient clinics were enrolled, and 18 incomplete responses were excluded, resulting in an analytic sample of 998. The total number of patients approached and the response rate were not formally recorded. NCDEG is a specialised diabetes centre and has individuals from all regions of Jordan, with a wide range of socioeconomic and demographic backgrounds, supporting the diversity of the study population. Data collection was performed using a questionnaire between April and October 2024 in the institution’s waiting hall, and they were invited to inquire if any question was unclear. The researchers read the questions for individuals who could not read. The inclusion criteria were individuals of any age and gender with type 1 or type 2 diabetes. To minimise selection bias, they were recruited in a consecutive manner during the study period.

Participants who declined to provide informed consent or individuals unable to complete the questionnaire due to severe cognitive impairment or acute illness at the time of recruitment were excluded from the study.

A structured questionnaire was designed based on similar studies [17,18,19]. The data collection tool was developed based on an extensive literature review and existing validated questionnaires. It was reviewed for content and face validity by a panel of five experts in endocrinology, ophthalmology, public health, and survey methodology. A pilot study (n = 50) was conducted to assess the clarity, feasibility, and comprehension of the questionnaire items; pilot responses were not included in the final analysis. Formal reliability testing (e.g., Cronbach’s alpha) was not performed. The questionnaire was developed in Arabic, the native language of participants, and was reviewed by two language experts to ensure clarity, cultural appropriateness, and accurate phrasing.

Standardised information about the identity of the researchers, the purpose of the study, how the data provided will be used, the estimated time to complete the questionnaire, voluntary participation, privacy, confidentiality, and anonymity of the data was explained to the participants before data collection, and they all consented before enrollment. The questionnaire was divided into four sections. The first section covered sociodemographic data, such as age, gender, living area, educational status, smoking and alcohol use, insurance, and whether they were employed in the healthcare sector or had a first-degree relative working in the medical sector. The second section covered their diabetes profile: the type of diabetes, duration since diagnosis, frequency of HbA_1c_ testing, their most recent and their highest HbA_1c_ level, and whether they had ever attended the ophthalmology clinic since their diagnosis. Based on their answer, the questionnaire would then move on to two different sections. Both sections included potential barriers to regular eye examination. However, those who had previously undergone an ophthalmic examination would also be asked about examination frequency, prior diagnosis of diabetic retinopathy, and if so, what treatment they have received. Additionally, participants were given the opportunity to provide their own perceived barriers which had not been captured by the questionnaire. All variables, including clinical information such as HbA_1c_ levels, history of diabetic retinopathy, and prior ophthalmic treatments, were self-reported by the respondents, and no data were extracted from medical records. Responses of “I don’t know” were treated as a valid response category. HbA_1c_ levels were reported as derived NGSP units (%—one decimal) in accordance with the unit used in NCDEG, which was then converted to IFCC units (mmol/mol). The primary outcome variable was history of having undergone an eye examination for diabetic retinopathy (ever vs. never), while the analysis primarily focused on identifying sociodemographic and participant-reported barriers associated with non-attendance or delayed eye examination (see Supplementary Material for the questionnaire).

The Statistical Package for the Social Sciences (SPSS) version 21.0 (IBM Corp., Armonk, NY, USA) was used for statistical analysis. Frequencies and percentages were used to convey qualitative data, whereas means and standard deviations (SDs) were used to summarise quantitative variables. The χ^2^ test assessed associations between categorical variables, and *p* values < 0.05 were considered statistically significant.

Associations of age, gender, education, income, insurance status, type and duration of diabetes, HbA_1c_ levels, and family or personal healthcare employment were compared between those who attended and those who did not attend eye clinic appointments. HbA_1c_ levels were categorised as within or above recommended targets in accordance with the recommendation of the American Diabetes Association (ADA) [20]. Variables showing clinical relevance or statistical significance in bivariate analyses were entered into a multivariable logistic regression model to identify factors independently associated with having undergone an eye examination. Results are reported as adjusted odds ratios (aORs) with 95% confidence intervals (CIs). To address small cell sizes, categories with low frequencies were combined where appropriate, and variables with sparse distributions were excluded from the multivariable model. No additional subgroup or sensitivity analyses were performed.

## 3. Results

### 3.1. Sociodemographic Characteristics of Participants

The study had 998 participants. As shown in Table 1, the majority were over 50 years old (82%). Most participants resided in urban areas and had a school-level education or higher. A significant proportion were non-smokers, and nearly all participants reported no alcohol consumption (99%). The majority had health insurance (97%) and 70% reported an income of less than JOD 1000.

### 3.2. Diabetes Profile

Table 2 demonstrates that the majority of participants had type 2 diabetes (79%). A significant portion (79%) had been living with diabetes for over 5 years. Most participants reported testing their HbA_1c_ levels every 1–3 months. The last recorded HbA_1c_ levels were mainly between 6 and 8%, with 14% reporting levels above 9%. A total of 70% had at least one ophthalmic examination since their diagnosis, and 27% of those had a screening interval of more than a year.

### 3.3. Diagnosis and Treatment of Diabetic Retinopathy

Table 3 shows that among the participants who had previously attended the ophthalmology clinic, 18% were diagnosed with diabetic retinopathy. Of these, 82% received laser-based treatment, retinal injections, or both.

### 3.4. Barriers to Having an Eye Examination Among Those Never Screened

The primary barrier to diabetic retinopathy screening among individuals never screened was lack of knowledge and perception, as shown in Table 4, with 56% reporting that their doctor did not mention it, and 62% believing that being asymptomatic negates the need for attendance. Almost one third of the patients reported logistical barriers such as transportation difficulties, and 59% reported multiple barriers.

### 3.5. Factors Contributing to the Delay in Regular Eye Examinations

For participants with prior eye examination, Table 5 displays that the majority (54%) believed that they were committed to attending appointments at the time they were surveyed. However, they were asked to mention if there are any factors that could hinder their commitment regardless. The most common factors contributing to the delay were knowledge and perception barriers, such as lack of awareness about the importance of regular reviews, and thinking that being asymptomatic eliminates the need for regular examinations. This was also followed by logistical barriers. Notably, 28% of participants cited more than one reason for the delay.

### 3.6. Problems Encountered During Eye Examinations

Table 6 illustrates that the majority of participants who had previously undergone an eye examination reported no issues. However, the most common reported problems were long waiting times, discomfort or inability to drive after dilation drops, and discomfort with the examination itself. A subset of participants (13%) encountered multiple problems.

### 3.7. Effect of Sociodemographic Characteristics and Diabetic Profile on Diabetic Retinopathy Screening (n = 998)

When examining the sociodemographic characteristics associated with having undergone at least one eye examination since diagnosis, Table 7 demonstrates that in bivariate analysis, age > 50 years, higher educational level, availability of health insurance, longer duration of diagnosis of diabetes mellitus, and HbA_1c_ > 7% were significantly associated with prior screening (*p* < 0.05).

### 3.8. Multivariable Logistic Regression Analysis of Factors Associated with Having Had a Previous Eye Examination (n = 998)

In multivariable logistic regression analysis (Table 8), older age (>50 years) was independently associated with lower odds of having had a previous eye examination (OR = 0.54; 95% CI: 0.31–0.96; *p* = 0.034), while male gender increased the odds (OR = 1.40; 95% CI: 1.06–1.85; *p* = 0.019). Participants without insurance were significantly less likely to have undergone an eye examination compared with those with insurance (OR = 0.31; 95% CI: 0.15–0.65; *p* = 0.002).

## 4. Discussion

There is an increase in the number of people being diagnosed with diabetes worldwide. As a result, a rise in eye complications associated with diabetes, such as diabetic retinopathy and diabetic macular oedema, is expected [21]. 

The majority of the participants had at least one prior examination, which aligns with a study from Saudi Arabia [17]. Only 122 (18%) respondents were diagnosed with diabetic retinopathy. This demonstrates significant differences compared to data collected from Jordan in 2008, where 64% of participants exhibited an element of diabetic retinopathy [22]. This difference could be explained by the underdiagnosis of diabetic retinopathy or self-reporting bias especially in the early stages, when the management is conservative.

Both a lack of awareness and believing that attendance should be related to symptoms could be associated with gaps in education at the time of diagnosis. When compared to other barriers, these had the highest percentages. Recommendations from healthcare professionals could act as an enabler to regular screening [23]. Accordingly, more care should be directed towards ensuring proper person–physician communication and education at the time of diagnosis. This lack of knowledge correlates with the results of a systematic review published in 2019, which found that the most consistent deterrent to screening across the majority of studies was a lack of knowledge about diabetic retinopathy [24]. On the other hand, some participants had an element of knowledge of diabetic retinopathy as a complication, but it was inaccurate and had been obtained from unreliable sources, such as social media, relatives, or friends. Some were also not convinced of the negative impact that diabetes can have on vision. Thus, it is crucial that they obtain their knowledge from trusted resources, which is also a necessary measure to consider.

Subsequently, other perceived barriers included those that made it difficult to obtain an appointment or reach a centre, such as transportation difficulties, unavailability of nearby centres, lack of insurance, unavailability of appointments, or difficulty booking an appointment. Similar results were noted in a 2013 published study, where transportation limited access to healthcare [25]. Also, lack of insurance was associated with reduced access to healthcare (*p* = 0.001). In the USA, before and after the implementation of the Affordable Care Act (ACA), the cost acting as a barrier to medical care decreased from 9.6% to 7% in adults [26]. A similar approach could be utilised in Jordan.

Moreover, only a minority of the participants had difficulties finding time off from work/lacked a companion to aid them in attending the appointment or were anxious about the exam results. This contradicts the findings reported in a study conducted in the UK, where its results illustrated that approximately one-third of participants had difficulties finding time off work or study to attend their appointments, 74.3% were worried that they may have diabetic retinopathy, and 63% were anxious about receiving their screening results [18].

Most of the participants that previously attended the ophthalmology clinic had no issues with it, suggesting that the service provided is good. However, discomfort following the administration of dilator drops, discomfort during the examination itself, or a long waiting time were the main issues faced. This aligns with the findings reported in the study conducted in the UK, where 64% complained about the adverse effects of the eye drops. Furthermore, those who did not attend regularly were more likely to complain that appointments occupied a significant portion of their day [18]. Some also were dissatisfied by the quality of the healthcare services (mistreatment by the staff/not receiving adequate time/not being seen by a specialist), which was associated with lower compliance. This was previously suggested by a systematic review published in 2013 that showed the negative impact of neglect on health outcomes [27].

According to multivariate analyses, younger age was independently associated with higher odds of prior eye examination; this could imply that they are more educated about the positive impact that regular reviews have on their overall well-being, which is similar to a recent study conducted in 2025 that showed that younger age groups had a superior health literacy [28]. According to bivariate analysis, having a higher level of education was associated with superior health awareness. Yet, this was expected, as it had been previously suggested [29]. Last but not least, participants with a previous level of HbA_1c_ above 7% were more likely to have had a previous ophthalmic examination, which may reflect the referral patterns observed in clinical practice when glycaemic control is suboptimal.

Our study has several limitations that should be acknowledged. First, the study relied on questionnaire-based data. While this is effective for gathering large amounts of data, it might lack depth and is susceptible to various biases, including social desirability and self-reported and recall biases. These biases could potentially affect the validity and accuracy of the findings, as well as the reliability of participants’ responses, which could lead to the underestimation of the diabetic retinopathy prevalence in patients. Second, some patients may have inaccurately reported previously receiving intravitreal injections or laser photocoagulation for diabetic retinopathy, as these treatment methods could also be performed for other retinal disorders. To address these weaknesses, future research should incorporate alternative data collection methods, such as objective assessments. Despite these limitations, the study has multiple strengths. One key strength is that it combines barriers to eye examination that have been previously reported in multiple other studies conducted outside of Jordan into a single framework tailored to the context of Jordanians with diabetes. Moreover, we acknowledge that a hospital-based design may have limitations in capturing community-level barriers. The study was conducted at NCDEG outpatient clinics, which serve patients from different regions of Jordan and include individuals across a wide range of socioeconomic and demographic backgrounds. This setting allowed us to recruit a large and diverse sample while ensuring feasibility and data quality, with adequate statistical power for subgroup analyses.

## 5. Conclusions and Recommendations

This study identified several key barriers to regular diabetic retinopathy screening among individuals with diabetes in Jordan. Most notably, knowledge and perception barriers, which included lack of awareness about the need for screening and the belief that the absence of symptoms negates the need for examination, were the most reported barriers. Other reported barriers were logistical, financial, and personal.

The most prevalent barriers to diabetic retinopathy screening in Jordan are largely modifiable and primarily related to low awareness and accessibility. Therefore, public health strategies should focus on addressing these barriers to enhance healthcare for individuals with diabetes.

Based on our findings, targeted interventions focusing on patient education at the time of diabetes diagnosis, improved physician–patient communication, and addressing logistical barriers such as transportation and appointment accessibility may enhance adherence to the recommended screening practices.

## Figures and Tables

**Table 1:** Sociodemographic characteristics of participants (*n* = 998).

Variable	*n* (%)
**Age (years)**	
Less than 30	55 (6)
30 to 50	125 (13)
More than 50	818 (82)
**Gender**	
Men	477 (48)
Women	521 (52)
**Living area**	
City	713 (71)
Out of the city	285 (29)
**Educational level**	
Illiterate	43 (4)
School	463 (46)
Bachelor’s or diploma	405 (41)
Postgraduate (master’s or PhD)	87 (9)
**Smoking**	
Non-smoker	700 (70)
Smoker	298 (30)
**Alcohol**	
Yes	8 (1)
No	990 (99)
**Insurance**	
Yes	968 (97)
No	30 (3)
**Income (JOD; GBP equivalent in parentheses)**	
Less than 1000 (1050)	702 (70)
1000 to 3000 (1050 to 3150)	275 (28)
More than 3000 (more than 3150)	21 (2)
**Are you employed in the healthcare sector?**	
Yes	55 (6)
No	943 (95)
**Do you have a first-degree relative working in the healthcare sector?**	
Yes	378 (38)
No	620 (62)

Data are presented as *n* (%). Abbreviations: JOD: Jordanian dinar; PhD: Doctor of Philosophy.

**Table 2:** Diabetes profile (*n* = 998).

Variable	*n* (%)
**Type of diabetes**	
Type 1 diabetes	113 (11)
Type 2 diabetes	786 (79)
I don’t know	99 (10)
**Duration since diagnosis with diabetes**	
Less than 5 years	212 (21)
Between 5 and 15 years	384 (39)
More than 15 years	402 (40)
**How often do you test your HBA_1c_ levels?**	
Every 1 to 3 months	906 (91)
Every 4 to 6 months	49 (5)
Every 7 to 12 months	28 (3)
Every more than 12 months	15 (2)
**Last HBA_1c_ mmol/mol (%)**	
31 to 42 (5.0 to 6.0)	114 (11)
42 to 53 (6.0 to 7.0)	289 (29)
53 to 64 (7.0 to 8.0)	296 (30)
64 to 75 (8.0 to 9.0)	156 (16)
More than 75 (more than 9.0)	135 (14)
I don’t know	8 (1)
**Highest HBA_1c_ mmol/mol (%)**	
31 to 42 (5.0 to 6.0)	14 (1)
42 to 53 (6.0 to 7.0)	97 (10)
53 to 64 (7.0 to 8.0)	139 (14)
64 to 75 (8.0 to 9.0)	147 (15)
More than 75 (more than 9.0)	589 (59)
I don’t know	12 (1)
**Have you had an ophthalmic examination since you were diagnosed with diabetes?**	
No	299 (30)
Yes	699 (70)
**How often do you have an eye examination?**	
Every 1 to 3 months	148 (21)
Every 4 to 6 months	181 (26)
Every 7 to 12 months	183 (26)
Every more than 12 months	187 (27)

Data are presented as *n* (%), Abbreviations: HBA_1c_: haemoglobin A_1c_.

**Table 3:** Diagnosis and treatment of diabetic retinopathy.

Are you diagnosed with diabetic retinopathy?	*n* (%)
Yes	**122 (18)**
No	**577 (83)**
**Have you received any treatment for diabetic retinopathy?**	
No, only conservative treatment	**22 (18)**
Retinal injection	**33 (27)**
Laser-based treatment	**21 (17)**
Retinal injection and laser-based treatment	**46 (38)**

Data are presented as *n* (%).

**Table 4:** Barriers to having an eye examination among those never screened (*n* = 299).

What Is the Reason for Not Visiting an Ophthalmologist?	*n* (%)
Knowledge and Perception Barriers	
**I was unaware that an eye examination is necessary when diagnosed with diabetes, as my doctor did not mention it.**	
Yes	167 (56)
No	132 (44)
**I am not fully convinced of the impact that diabetes has on the eye and retina.**	
Yes	23 (8)
No	276 (92)
**I am not experiencing any symptoms; thus, I believe that there is no need to attend the ophthalmology clinic.**	
Yes	186 (62)
No	113 (38)
Access and Financial Barriers	
**I do not have insurance or an exemption, which makes it harder for me to access the necessary care.**	
Yes	31 (10)
No	268 (90)
**There is no medical centre nearby where I can get an eye examination.**	
Yes	19 (6)
No	280 (94)
Logistical Barriers	
**Difficulty with transportation has caused a delay in scheduling the eye examination.**	
Yes	38 (13)
No	261 (87)
**Due to difficulty obtaining leave from work or a lack of a companion to assist with the medical visit, I have been unable to schedule the eye examination.**	
Yes	17 (6)
No	282 (94)
**Difficulty booking an appointment.**	
Yes	16 (5)
No	283 (95)
**There are no appointments available soon.**	
Yes	13 (4)
No	286 (96)
Psychosocial Barriers	
**I have a fear of being diagnosed with something more serious and being required to take additional medications.**	
Yes	20 (7)
No	279 (93)
**I have encountered misinformation or discouragement from social media, friends, or relatives.**	
Yes	23 (8)
No	276 (92)
Personal Factors	
**Lack of time due to social obligations, health concerns, and travel has prevented me from scheduling the eye examination.**	
Yes	23 (8)
No	276 (92)
**I lack the motivation to follow through with appointments, whether it’s due to laziness, forgetting the scheduled time, or general negligence in managing my health.**	
Yes	14 (5)
No	285 (95)
**I was diagnosed with diabetes only recently.**	
Yes	4 (1)
No	295 (99)
**More than one reason.**	**177 (59)**

Data are presented as *n* (%).

**Table 5:** Factors contributing to the delay in regular eye examinations (*n* = 699).

What Are Common Barriers You May Face that Delay Regular Eye Examinations?	*n* (%)
**I believe I am committed to attending appointments regularly**	
Yes	380 (54)
No	319 (46)
Knowledge and Perception Barriers	
**I was unaware of the importance of regular reviews, as my doctor did not inform me of them.**	
Yes	79 (11)
No	620 (89)
**I am not fully convinced of the impact that diabetes has on the eye and retina.**	
Yes	14 (2)
No	685 (98)
**I am not experiencing any symptoms; thus, I believe that there is no need to attend the ophthalmology clinic.**	
Yes	134 (19)
No	565 (81)
Access and Financial Barriers	
**I do not have insurance or an exemption, which makes it harder for me to access the necessary care.**	
Yes	34 (5)
No	665 (95)
**There is no medical centre nearby where I can get an eye examination.**	
Yes	54 (8)
No	645 (92)
Logistical Barriers	
**Difficulty with transportation has caused a delay in scheduling the eye examination.**	
Yes	95 (14)
No	604 (86)
**Due to difficulty obtaining leave from work or a lack of a companion to assist with the medical visit, I have been unable to schedule the eye examination.**	
Yes	35 (5)
No	664 (95)
**Difficulty booking an appointment.**	
Yes	27 (4)
No	672 (96)
**There are no appointments available soon.**	
Yes	39 (6)
No	660 (94)
Psychosocial Barriers	
**I have a fear of being diagnosed with a more serious condition and being required to take additional medications.**	
Yes	13 (2)
No	686 (98)
**I have encountered misinformation or discouragement from social media, friends, or relatives.**	
Yes	11 (2)
No	688 (98)
Personal Barriers	
**Lack of time due to social obligations, health concerns, and travel has prevented me from scheduling the eye examination.**	
Yes	77 (11)
No	622 (89)
**I lack the motivation to follow through with appointments, whether it’s due to laziness, forgetting the scheduled time, or general negligence in managing my health.**	
Yes	26 (4)
No	673 (96)
**More than one reason**	**198 (28)**

Data are presented as *n* (%).

**Table 6:** Problems encountered during eye examinations (*n* = 699).

Problems	*n* (%)
No problem	469 (67)
Waiting time length	121 (17)
Inability to drive after the dilator drop or my discomfort with it	119 (17)
Mistreatment by health staff	17 (2)
My discomfort with the examination itself	70 (10)
Not giving me enough consultation time	12 (2)
Not being seen by specialists (seen by students, trainees, or residents)	4 (1)
More than one problem	93 (13)

Data are presented as *n* (%).

**Table 7:** Effect of sociodemographic and diabetic profile on diabetic retinopathy screening (*n* = 998).

Variable	Category	Previously Had an Eye Examination *n* (%) 699 (70)	Never Had an Eye Examination *n* (%) 299 (30)	Total *n* (%)	*p*-Value
**Age (years)**	Less than 30	33 (60)	22 (40)	55 (6)	0.003
	30 to 50	74 (59)	51 (41)	125 (13)	
	More than 50	592 (72)	226 (28)	818 (82)	
**Gender**	Men	348 (73)	129 (27)	477 (48)	0.054
	Women	351 (67)	170 (33)	521 (52)	
**Educational Level**	Illiterate	27 (63)	16 (37)	43 (4)	0.037
	School	310 (67)	153 (33)	463 (46)	
	Bachelor’s or diploma	292 (72)	113 (28)	405 (41)	
	Postgraduate	70 (81)	17 (20)	87 (9)	
**Income (JOD)**	Less than 1000	486 (69)	216 (31)	702 (70)	0.447
	1000 to 3000	196 (71)	79 (29)	275 (28)	
	More than 3000	17 (81)	4 (19)	21 (2)	
**Living Area**	City	500 (70)	213 (30)	713 (71)	0.925
	Out of the city	199 (70)	86 (30)	285 (29)	
**Insurance**	Yes	686 (71)	282 (29)	968 (97)	0.001
	No	13 (43)	17 (57)	30 (3)	
**Healthcare Worker**	Yes	37 (67)	18 (33)	55 (6)	0.645
	No	662 (70)	281 (30)	943 (95)	
**First-Degree Relative in the Healthcare Sector**	Yes	278 (74)	100 (27)	378 (38)	0.059
	No	421 (68)	199 (32)	620 (62)	
**Type of Diabetes**	Type 1	76 (67)	37 (33)	113 (11)	0.649
	Type 2	556 (71)	230 (29)	786 (79)	
	I don’t know	67 (68)	32 (32)	99 (10)	
**Duration Since Diagnosis**	Less than 5 years	96 (45)	116 (55)	212 (21)	<0.001
	5 to 15 years	267 (70)	117 (31)	384 (39)	
	More than 15 years	336 (84)	66 (16)	402 (40)	
**HbA_1c_ Level mmol/mol (%)**	Less than 53 mmol/mol (less than 7%) (within recommended target)	256 (64)	147 (37)	403 (40)	<0.001
	More than or equals to 53 mmol/mol (more than or equal to 7%) (above recommended target)	440 (75)	147 (25)	587 (59)	
	Don’t know	3 (38)	5 (63)	8 (1)	

*p*-values derived from chi-square tests for categorical variables. Data are presented as *n* (%). Statistical tests: chi-square for categorical variables; all *p*-values are two-tailed. Abbreviations: JOD: Jordanian dinar; HbA_1c_—haemoglobin A_1c_.

**Table 8:** Multivariable logistic regression analysis of factors associated with having had a previous eye examination (*n* = 998).

Variables	OR	95% CI	*p*-Value
**Age (years)**			0.003
<30 *	1	—	—
30–50	0.97	0.50–1.86	0.922
>50	0.54	0.31–0.96	0.034
**Gender**			0.019
Women *	1	—	—
Men	1.40	1.06–1.85	0.019
**Insurance status**			0.002
Yes *	1	—	—
No	0.31	0.15–0.65	0.002

* Reference group.

## Data Availability

The data supporting this study are not publicly available due to patient confidentiality, but they are available from the corresponding author on reasonable request; details of the applicable restrictions and access conditions will be provided as appropriate.

## Supplementary Materials

The following supporting information can be downloaded at https://www.mdpi.com/article/10.3390/ijerph23020147/s1.

## Abbreviations

The following abbreviations are used in this manuscript:

MENA	Middle East and North Africa
NPDR	Non-Proliferative Diabetic Retinopathy
PDR	Proliferative Diabetic Retinopathy
ETDRS	Early Treatment Diabetic Retinopathy Study
NCDEG	National Center for Diabetes, Endocrinology and Genetics
IRB	Institutional Review Board
HbA_1c_	Haemoglobin A_1c_
SPSS	Statistical Package for the Social Sciences
SD	Standard Deviation
ACA	Affordable Care Act
