# Supplementary material for: Barriers to Regular Eye Examination in Individuals with Diabetes at a Tertiary Diabetes Centre in Jordan: A Cross-Sectional Study
Source: Int J Environ Res Public Health. 2026 Jan 24;23(2):147. doi: 10.3390/ijerph23020147 (PMC12941273; doi:10.3390/ijerph23020147)
Supplement: Supplementary file 1 [file ijerph-23-00147-s001.zip › ijerph-4070942-supplementary.pdf]

TRANSLATED QUESTIONNAIRE

Consent

This research is being conducted by a group of medical students and physicians from the University of Jordan and the National Center for Diabetes, Endocrinology and Genetics. It aims to study diabetic retinopathy, a retinal disease associated with diabetes, and the factors that may affect the speed of diagnosis and prevention of this condition, and identify the sociodemographic, personal, and institutional barriers that affect the adherence to regular screening among people with diabetes.

We guarantee complete confidentiality of the information and that it will not be used for any purpose outside the scope of this research. There is no risk or harm to participating individuals, and you have the right to withdraw from the study at any time.

Consent to participate

(Mark only one)

• Agree

Demographic and Social Background

1. Age group

(Mark only one)

• Less than 30 years

• 30 to 50 years

• More than 50 years

2. Type of diabetes

(Mark only one)

• Type 1

• Type 2]

• I don’t know

3. Gender

(Mark only one)

• Male

• Female

4. Smoking status

(Mark only one)

• Smoker

• Non-smoker

5. Alcohol consumption

(Mark only one)

• Yes

• No

6. Educational level

(Mark only one)

• Illiterate

• School education

• Bachelor’s degree or Diploma

• Postgraduate studies (Master’s / PhD)

7. Do you work in the healthcare sector?

(Physician, nurse, pharmacist, physical therapist)

(Mark only one)

• Yes

• No

8. Do you have a first-degree relative working in the healthcare sector?

(Brother/sister, spouse, children, father, mother)

(Mark only one)

• Yes

• No

9. Place of residence

(Mark only one)

• City

• Outside the city

10. Monthly family income(JD)

(Mark only one)

• Less than 1000

• 1000 to 3000

• More than 3000

11. Do you have health insurance / exemption?

(Mark only one)

• Yes

• No

General Information

12. When were you diagnosed with diabetes?

(Mark only one)

• Less than 5 years ago

• Between 5 to 15 years ago

• More than 15 years ago

13. Last HbA1c reading

(Mark only one)

• 5–6

• 6–7

• 7–8

• 8–9

• More than 9

• I don’t know

14. Highest HbA1c reading

(Mark only one)

• 5–6

• 6–7

• 7–8

• 8–9

• More than 9

• I don’t know

15. How often do you check HbA1c?

(Mark only one)

• Every 1-3 months

• Every 4-6 months

• Every 7–12 months

• Every more than 12 months

Ophthalmology Clinic Attendance

16. Is this the first time you visit an ophthalmology clinic after being diagnosed with diabetes?

(Mark only one)

- Yes, this is the first time → Skip to Question 17
- No, not the first time → Skip to Question 18
- I have never visited an eye clinic since diagnosis → Skip to Question 17

Barriers to First Eye Examination

17. What prevented you from attending an eye examination earlier?

(Tick all that apply)

- I was not aware of the need for an eye examination at diagnosis (the doctor did not inform me)
- Not convinced that diabetes affects the eyes and retina
- Lack of insurance or exemption
- No nearby center
- Transportation difficulties
- Difficulty obtaining leave or lack of a companion
- Fear of diagnosis and being obligated to additional medications
- Difficulty booking an appointment
- No nearby available appointments

• Misinformation or lack of encouragement from social media, friends, or relatives

• No symptoms

• Other commitments (social, health-related, etc.)

• Others

Barriers to Regular Eye Examination

18. How often do you undergo retinal examination?

(Mark only one)

• Every 1-3 months

• Every 4-6 months

• Every 7–12 months

• Every more than 12 months

19. What prevented you from undergoing regular eye examinations?

(Tick all that apply)

• I adhere to appointments

• I was not aware of the importance of regular follow-up (doctor did not inform me)

• Not convinced that diabetes affects the eyes and retina

• Lack of insurance or exemption

• No nearby center

• Transportation difficulties

• Difficulty obtaining leave or lack of a companion

• Fear of diagnosis and being obligated to additional medications

- Difficulty booking appointments
- No nearby available appointments
- Misinformation or lack of encouragement from social media, friends, or relatives
- No symptoms
- Others

20. What problems did you face during the eye examination?

(Tick all that apply)

- Long waiting time
- Inability to drive after dilating drops or discomfort from them
- Poor behavior of healthcare staff
- Discomfort from the examination itself
- None
- Others

21. Have you been diagnosed with diabetic retinopathy?

(Mark only one)

- Yes → Skip to Question 25
- No

For Patients Diagnosed with Diabetic Retinopathy

22. Have you received any treatment for diabetic retinopathy?

(Tick all that apply)

- Retinal injections
- Laser treatment
- No, conservative treatment only
